# Does the number of apoptotic cells in PBMC reveal the extent of impairment in T cell functionality?

**DOI:** 10.1186/2051-1426-1-S1-P110

**Published:** 2013-11-07

**Authors:** Srividya Sundararaman, Marie Wunsch, Richard Caspell, Paul V Lehmann

**Affiliations:** 1 R&D, Cellular Technology Ltd., Shaker Hts., OH, USA

## 

As soon as PBMC are isolated cells start dying. The rate of apoptotic cell death increases when PBMC are shipped, cryopreserved or stored under suboptimal conditions. Apoptotic cells secrete mediators such as Lactoferrin that suppress inflammation while promoting phagocytosis. The presence of apoptotic cells in PBMC may modulate functionality in antigen-specific T cell assays such as ELISPOT or ICS. Also, apoptotic cells in PBMC indicates damage to the sample, and may show deficient T cell functions that affect non-apoptotic T cells as well. In one approach anti-CD20 MAb-coupled beads were used to selectively induce apoptosis in the B cell subpopulation of PBMC followed by ELISPOT testing. In a second approach supernatants of purified B cells undergoing apoptosis were added to PBMC and tested in ELISPOT assays. In a third approach thawed PBMC were stored overnight at 4°C inducing an increased rate of apoptosis compared to PBMC stored in the incubator overnight. CD8 and CD4 cell reactivity were tested in an hIFN-γ ELISPOT assay. Live, dead and apoptotic cells were counted using CTL’s cell counting platform. Overnight treatment with anti-CD20 antibody induced apoptosis in close to 100% of the B cells. Storage of PBMC overnight at 4°C induced apoptosis in 6% of the PBMC. However, the apoptotic process continued for 24 hour in an ELISPOT assay resulting in an additional 17% apoptosis. Testing of the PBMC in which B cells were selectively induced to undergo apoptosis showed that the antigen- specific ELISPOT counts were unaffected. Supernatants of apoptotic B cells did not affect the ELISPOT counts. These data argue against mediators released by dying cells (in this case B cells) interfering with CD8 T cell functionality in ELISPOT assays. In contrast, testing of the overnight 4°C treated PBMC however showed that hIFN- γ response was reduced Apoptosis of bystander cells per se does not impact CD8 cell function. Measuring the number of apoptotic cells before plating the PBMC does not reflect the extent of cell injury. An increased rate of apoptosis continues in damaged cell samples throughout the subsequent T cell assay. Measuring the number of apoptotic cells during and after the T cell assay reveals damage to the PBMC. In addition to measuring live, dead and apoptotic cells in PBMC, additional markers are needed that would identify sublethal damages to the cells, and therefore help assess whether a PBMC sample is suitable for functional T cell assays.

